# TBCD Links Centriologenesis, Spindle Microtubule Dynamics, and Midbody Abscission in Human Cells

**DOI:** 10.1371/journal.pone.0008846

**Published:** 2010-01-22

**Authors:** Mónica López Fanarraga, Javier Bellido, Cristina Jaén, Juan Carlos Villegas, Juan Carlos Zabala

**Affiliations:** 1 Departamentos de Biología Molecular, Instituto de Formación e Investigación Marqués de Valdecilla Facultad de Medicina, Universidad de Cantabria, Santander, Spain; 2 Anatomía y Biología Celular, Instituto de Formación e Investigación Marqués de Valdecilla Facultad de Medicina, Universidad de Cantabria, Santander, Spain; Duke University Medical Centre, United States of America

## Abstract

Microtubule-organizing centers recruit α- and β-tubulin polypeptides for microtubule nucleation. Tubulin synthesis is complex, requiring five specific cofactors, designated tubulin cofactors (TBCs) A–E, which contribute to various aspects of microtubule dynamics in vivo. Here, we show that tubulin cofactor D (TBCD) is concentrated at the centrosome and midbody, where it participates in centriologenesis, spindle organization, and cell abscission. TBCD exhibits a cell-cycle-specific pattern, localizing on the daughter centriole at G1 and on procentrioles by S, and disappearing from older centrioles at telophase as the protein is recruited to the midbody. Our data show that TBCD overexpression results in microtubule release from the centrosome and G1 arrest, whereas its depletion produces mitotic aberrations and incomplete microtubule retraction at the midbody during cytokinesis. TBCD is recruited to the centriole replication site at the onset of the centrosome duplication cycle. A role in centriologenesis is further supported in differentiating ciliated cells, where TBCD is organized into “centriolar rosettes”. These data suggest that TBCD participates in both canonical and de novo centriolar assembly pathways.

## Introduction

Understanding how the centrosomal components function and are organized during the cell cycle could shed light on many human diseases, from ciliary syndromes to cancer. The centrosome is the major microtubule-organizing center (MTOC) in animal cells, consisting of two centrioles surrounded by proteinaceous pericentriolar material (PCM), from which microtubules nucleate and are released. Microtubules are built of αβ-tubulin heterodimers organized in a head-to-tail fashion. This confers polarity on these polymers, with two different microtubule ends: the plus end, which is more dynamic and oriented towards the periphery of the cells, and the minus end, generally embedded in the centrosome. At the centrosomal core, a centriole pair organizes the surrounding PCM. Centrioles, which are required to assemble the axonemes of cilia and flagella, are structurally very complex. In mammals, they are 500 nm long cylinders with a 200 nm diameter consisting of nine blades arranged in a circle, each containing three highly specialized microtubule segments. Both the exact composition and the assembly of these peculiar microtubules are still unknown. There is also imprecise information regarding the composition and structure of the PCM, in which recent studies have identified hundreds of proteins, most of which play as yet unknown roles [1]. However, one of the most widely accepted concepts is that MTOCs, and in particular the centrosome, accumulate αβ-tubulin polypeptides as part of the PCM for microtubule nucleation. This suggests that there is some tubulin supply at the MTOCs. Yet, the assembly of αβ-tubulin heterodimers is not a trivial matter. The process of association of one α-tubulin with one β-tubulin molecule requires the co-ordinated interaction of a series of tubulin-specific partners, designated tubulin cofactors (TBCs) A–E [2]–[5]. TBCs also play roles in tubulin dissociation [5]–[8], transitory tubulin storage [5], [9]–[12], and tubulin degradation processes [13]–[15], all of which suggest that these proteins, in addition to their original role in tubulin biogenesis, participate in microtubule dynamics by controlling the amount of tubulin available for polymerization. There is considerable evidence in the literature supporting a role for TBCs in this centrosome. Mutations in TBCD, in particular, have been shown to produce aberrations in chromosome numbers in Saccharomyces cerevisiae [16], Schizosaccharomyces pombe [17], Arabidopsis thaliana [18], [19], and Caenorhabditis elegans [20], [21]. TBCD mutations also induce a G1/S blockage and spindle pole body separation in S. pombe [22], [23] and abnormal cytokinesis [19]. In C. elegans, TBCD silencing results in a reduced rate of microtubule nucleation and produces abnormal spindle lengths [21]. Recently, human TBCD (HsTBCD) has been shown to play a role in the organization of the mitotic spindle, and has been hypothesized to recruit from among cytosolic centrosomal proteins, such as pericentrin or γ-tubulin [24].

Our data show that TBCD accumulates in immature centrioles and at the midbody ring during cytokinesis. We demonstrate TBCD recruitment into “centriolar rosettes” during basal-body assembly in a novel primary cell culture system of differentiating ciliated cells, which we developed for this study. More interestingly, we demonstrate that the manipulation of TBCD levels produces several abnormalities at the centrosome and midbody, resulting in spindle and anaphase defects, G1 blockage, and cell abscission failure. These findings indicate that TBCD is a fundamental protein in cell division.

## Results

### TBCD Is Concentrated in Centrioles and Midbodies

**Figure 1:**
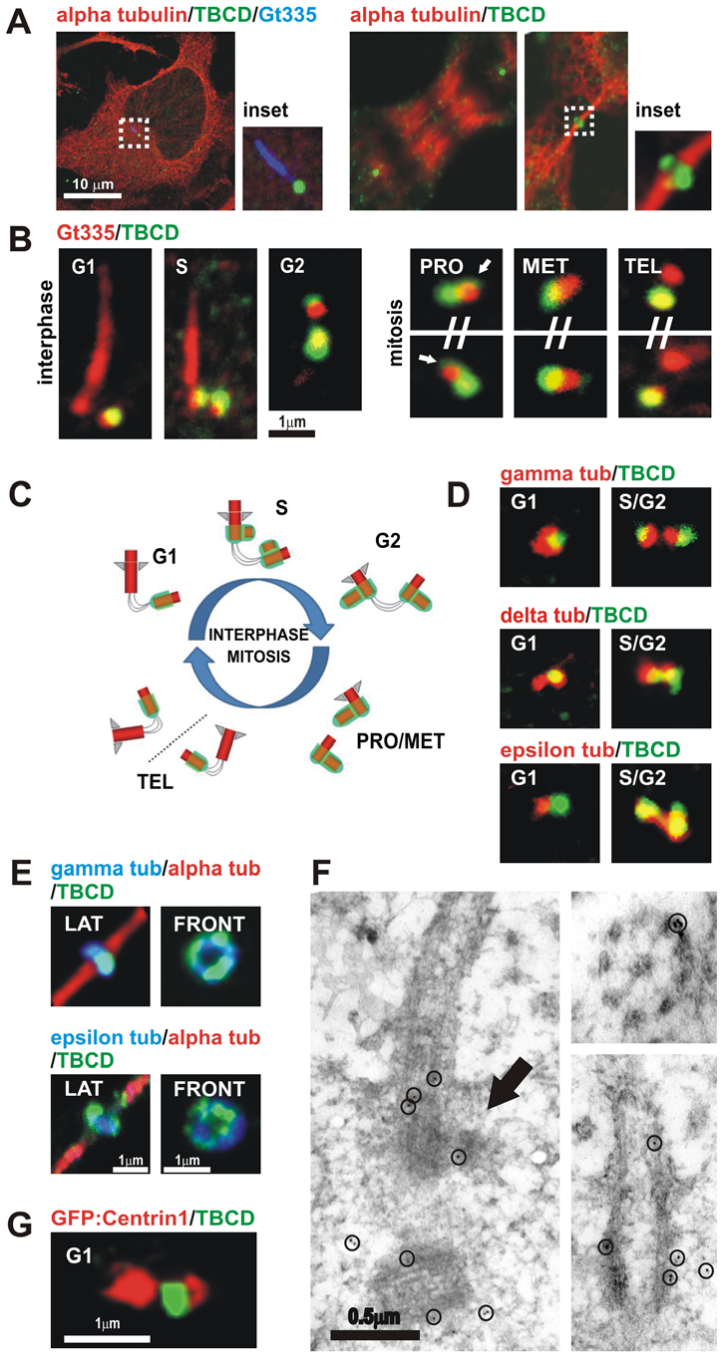
TBCD is concentrated in centrioles and midbodies. (A) Confocal-microscopic images of a HeLa cell (ATCC^R^ number: CCL-2™) immunostained for tubulin, TBCD, and GT335 (which labels glutamylated tubulin at the centrioles and primary cilium), where a single spot of TBCD is observed close to the basal body of the cilium. HeLa cells in anaphase and undergoing cytokinesis display clear centrosomal labelling and recruitment of TBCD to the midbody ring, respectively. (B) High-resolution confocal-microscopic section images of doubly immunostained centrosomes of HeLa cells. The cells were photographed at different stages of the cell cycle, labelled with GT335 and anti-TBCD antibody. This tubulin cofactor is observed at one of the centrioles during G1. Two stronger additional TBCD signals immediately adjacent to the existing centrioles, devoid of GT335 staining, were stained at G1. By late S three TBCD spots were detected. By prophase, most dividing cells contained two strong TBCD spots, one in each centrosome. Partial TBCD halos surrounding older centrioles were also observed (arrows). At the end of telophase, only one of the centrioles in each daughter cell was labelled for TBCD. (C) Diagram of the distribution of TBCD during the centriolar cycle; centrioles, procentrioles, and primary cilium are shown in red and TBCD is shown in green. (D) Partial co-localization of the TBCD signal with γ- and δ-tubulins was observed throughout the cell cycle. The ε-tubulin signal concentrated at the mother centriole during G1 did not co-localize with TBCD. Later in the cell cycle, both proteins appeared to partially co-localize. (E) TBCD accumulated at the midbody, where γ- and ε-tubulins were also detected. Lateral and frontal views of the structure correspond to different cells. (F) Immuno-electron-microscopic analysis of TBCD on murine epithelial cells. (Left) TBCD localises on the proximal region of the basal body (the original mother centriole) during procentriole assembly (arrow) S stage. The former daughter centriole is also observed in the section (gold particles are outlined with circles). (Right top) TBCD labelling was also detected at the proximal ends of basal bodies and (Right bottom) at the outer microtubule doublets of motile tracheal cilia. (G) Immunostaining of HeLa cells transfected with GFP-centrin1 (red, pseudocolor) confirm TBCD localization at the proximal end of the daughter centriole.

TBCD has been reported in the centrosome and has been shown to co-sediment with γ-tubulin when overexpressed [24], but the exact location of the protein has not been documented. To investigate more precisely its centrosomal distribution, we first produced and affinity purified several anti-TBCD antibodies (Figure S1A), which we used to identify the sub-cellular locations of this cofactor in HeLa cells, among other systems. We observed that despite all the biochemical evidence suggesting that TBCD is predominantly a cytoplasmic protein, TBCD accumulated prominently at the centrosome (Figure 1A), as determined by double immunostaining for standard markers, such as γ-tubulin, NEDD1/GCP-WD (Figure S1B), and others. Microtubule depolymerization by nocodazole and cold treatment showed that TBCD localization at the centrosome is not dependent on microtubules, thus demonstrating that TBCD behaves as a genuine centrosomal protein.

Using high-resolution confocal microscopy, we next investigated the relationship of these TBCD spots with the centrioles and primary cilium. For this purpose, we doubly/triply immunostained cells with our antibodies and combinations of various markers, including GT335, which is a broadly characterized antibody that recognizes glutamylated tubulin and is typically used to label centrioles and the primary cilium. We observed one TBCD spot accompanying the primary cilium in cells at G1, which we identified as occurring at the daughter centriole by double immunostaining with an antibody that recognizes ε-tubulin, which did not co-localize with TBCD (Figure 1B–D). As mitosis progressed, two stronger TBCD signals devoid of GT335 staining arose immediately adjacent to the existing centrioles. This suggests that TBCD is localized on developing procentrioles before tubulin glutamylation.

During late G2, although often four GT335 spots were visible (Figure 1B, C), only three TBCD spots of different intensities were observed. Two intense spots were localized on developing centrioles before tubulin glutamylation, and a weaker signal on the former daughter centriole. Sometime after cilia disassembly, the TBCD signal on the original daughter centriole weakened and by the beginning of mitosis, in prophase, most dividing cells already showed only two strong TBCD spots localized on the newest centriole of each pair. Occasionally, a TBCD halo surrounding older centrioles was also observed (Figure 1B, arrows), suggesting that this protein connects both the old and the new centrioles at this point. Partial co-localization of the TBCD signal with γ- and δ-tubulin was observed during the cell cycle. TBCD also appeared to co-localize with ε-tubulin in cells at late S/G2 phase (Figure 1D), coinciding with the reported redistribution of this tubulin [25]. By telophase, after centriole separation, only the new centriole of each pair was labelled for TBCD. This phenomenon coincided chronologically with the accumulation of TBCD in a ring-like structure localized at the midbody at the point of cell abscission (Figure 1A), where we also identified γ- and ε-tubulin (Figure 1E).

Immuno-gold localization of TBCD revealed proximal centriolar and procentriolar labelling, which was also conspicuous at the proximal end of the basal bodies of the cilia (Figure 1F), a pattern almost identical to that described for γ-tubulin [26]. TBCD labelling was also observed on ciliary microtubules. We further confirmed TBCD localization at the proximal end of centrioles immunostaining GFP-centrin1 transfected HeLa cells (Figure 1G).

The observed cell-cycle-specific pattern and the association of TBCD with procentrioles implicate this tubulin cofactor in centriologenesis. To explore this hypothesis further, we performed additional experiments in different systems. First, we analysed TBCD expression using immunofluorescence in Chinese hamster ovary (CHO) cells arrested at G1/S phase by thymidine treatment. This induces repeated cycles of centriole/centrosome synthesis, thus providing a convenient assay with which to identify the molecular components involved in centriologenesis. Our results confirmed the TBCD immunostaining on developing centrioles after G1 blockage (Figure S2A). An alternative method used to trigger massive centriologenesis is the overexpression of proteins such as PLK4 or SAS-6, which are among the first proteins reported in the recruitment of centriolar components [27], [28]. Therefore, we overexpressed PLK4 on HeLa cells and looked for TBCD clustering at the developing centrioles. This system also confirmed TBCD accumulation at the centrosome (Figure S2B).

### TBCD Overexpression Releases Microtubules from the Centrosome

**Figure 2:**
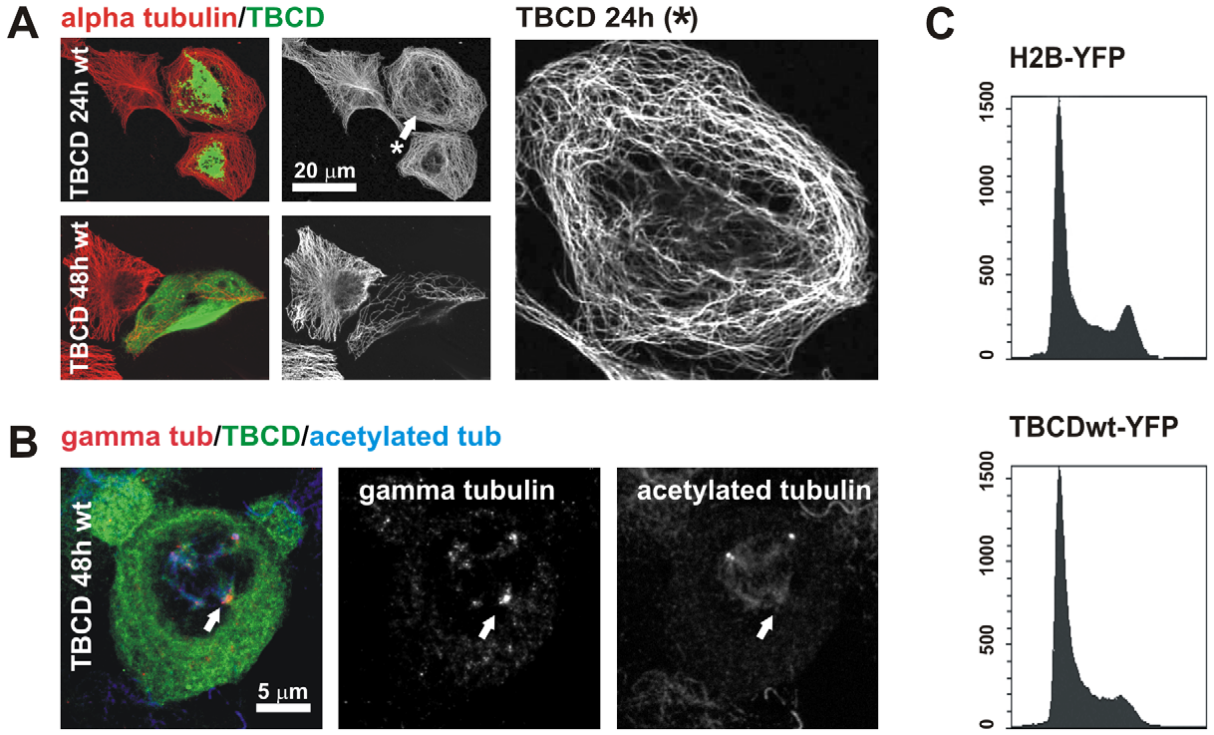
TBCD overexpression resulted in microtubule detachment from the centrosome and spindle abnormalities. (A) Confocal-microscopic projected images of a HeLa cell overexpressing HsTBCD doubly immunostained for tubulin and TBCD. (Top) Microtubule release from the centrosome was clear in most cells 15 h after transfection (arrow). (Bottom) Microtubule network destruction occurred approximately 30 h after transfection. Right panel shows a closer view of the microtubule pattern of the cell labelled with an arrow.(B) TBCD overexpression also produced aberrant mitotic figures, where acentriolar supernumerary MTOCs containing γ-tubulin were observed (arrow). (C) Flow-cytometric profiles of cells (>10,000 cells per profile) labelled with Hoechst and transfected with HsTBCD–YFP or a control–YFP construct. Clear G1 arrest, typical of the loss of centrosomal integrity, was observed in the cells analysed at both 15 and 30 h after transfection.

Human TBCD (HsTBCD) has been reported to behave differently when overexpressed to the bovine protein (BtTBCD) [24], which was the first TBCD isolated and has therefore been more often studied in vitro and in vivo [6], [8]. Consequently, we decided to investigate changes in the microtubule cytoskeleton organization in cells overexpressing these two orthologues. We observed that, compared with BtTBCD, HsTBCD was more condensed at the centrosome and the microtubule cytoskeleton took longer to depolymerise. Quantification of microtubule depolymerisation in over-expressing cells at 15 and 30 hours post-transfection revealed that although both proteins resulted in a massive microtubule destruction at 30 hours post-transfection (71% Hs Vs 83% Bt), HsTBCD overexpressing cells exhibited no obvious microtubule depolymerisation signs at 15 hours (100% cells contained microtubules) while 82% of cells overexpressing BtTBCD displayed a complete microtubule destruction. More interestingly, we found that HsTBCD accumulation appeared to detach the microtubule minus ends from the centrosome (Figure 2A). This finding can be understood in view of previous observations that overexpressed HsTBCD removes γ-tubulin from the centrosome [24] and suggests that this tubulin cofactor could play a role in microtubule release in γ-tubulin-dependent MTOCs.

We also observed that TBCD overexpression produced aberrant mitotic figures, where supernumerary MTOCs containing γ-tubulin, but no detectable centrioles, developed (Figure 2B). These observations suggest that there is a delay at mitosis, which we next investigated by flow cytometry in cells transfected with constructs encoding HsTBCD–yellow fluorescent protein (YFP) or H2B–YFP as the control. Flow cytometry analysis 15 h post-transfection revealed that 65% of TBCD positive cells were found at G1 compared to 45% of controls. Hence, we conclude that TBCD overexpression results in G1 delay (Figure 2C). This finding is consistent with those described in S. pombe, where mutations in the TBCD orthologue, Alp1p, produced a G1/S block [22]–[23]. In view of recent reports demonstrating that centrosomal integrity is required for G1/S progression [29], these findings support the notion that TBCD is a key centrosomal protein.

### TBCD Contains a Microtubule-Binding Region and Two Centriolar-Targeting Regions

**Figure 3:**
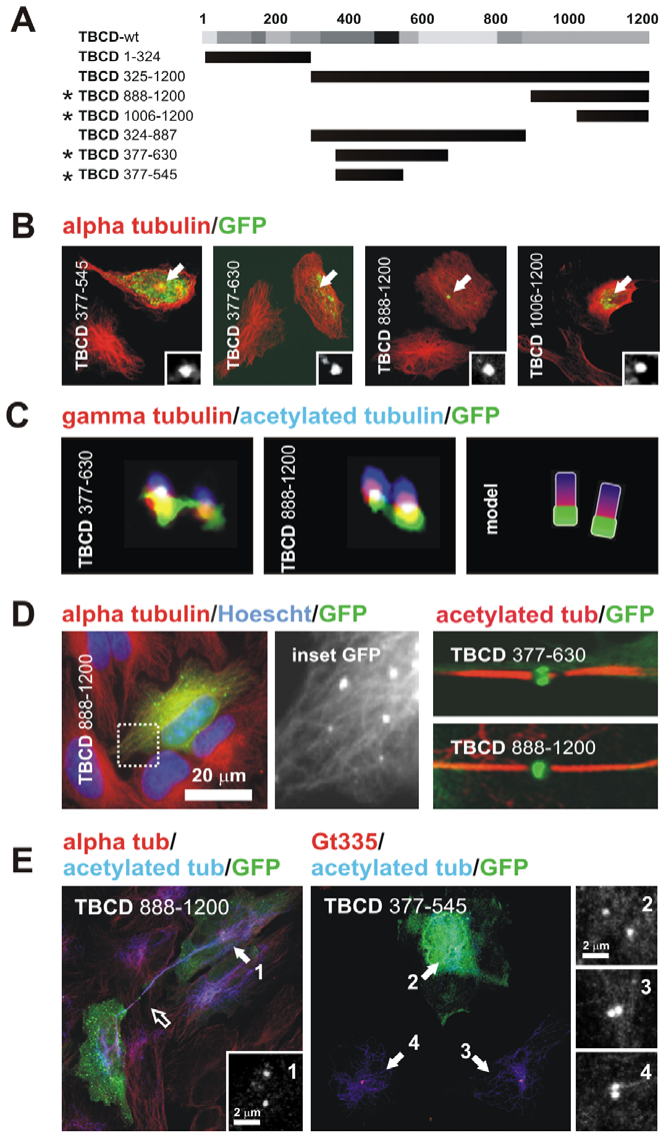
TBCD contains a microtubule-binding region and two centriolar-targeting regions. (A) Diagram of the full-length TBCD polypeptide and the truncation mutants produced for this study. The degree of evolutionary conservation of the polypeptide is shown in relative shades of grey. (B) Confocal images of HeLa cells transfected with constructs encoding GFP-fusion TBCD truncation mutants and immunostained for tubulin. The constructs shown correspond to those labelled with an asterisk in A. The GFP labelling at the centrosomal region is shown in the inset. (C) High-resolution confocal images of triply labelled centrioles in weakly expressing cells show the co-localization of these two TBCD truncation mutants with γ-tubulin in the proximal region of both centrioles. (D) TBCD contains a microtubule-binding region. GFP–TBCD_888–1200_-decorated microtubules 48 h after transfection. (Left) TBCD centrosomal-binding truncation mutants were also recruited to the Fleming bodies of cells at the end of mitosis. (E) Phenotypes observed for TBCD centrosomal-binding truncation mutants. Aberrant midbodies with increased lengths and reduced thicknesses were common (right, open arrow). Centriolar separation was increased (>2 µm) in many of the transfected cells (centrosomes 1, 2 versus 3, 4).

We next undertook to localize the centriolar-targeting region in the TBCD polypeptide. Bio-informatic analysis of different TBCD sequences revealed two segments in the polypeptide, extending approximately from amino acids (aas) 350 to 550 and from aa 1000 to the C-terminus, with a significant degree of evolutionary conservation (over 30% and 21% identity between mammals and yeast, respectively, for the first region, and over 10% for the second region in both organisms). We next generated constructs encoding these TBCD segments (aas 377–545 and aas 1006–1200) by PCR and cloned them for transitory transfection into HeLa cells for in vivo study. This study was complemented with other polypeptide segments, including aas 1–324, 325–1200, 324–887, 377–630, and 888–1200, of the BtTBCD sequence (Figure 3A). The study revealed that the two conserved TBCD protein segments were directed to the centrosome, co-localizing with various centrosomal markers (data not shown). Whereas none of these constructs caused microtubule depolymerization, microtubule pattern changes at the centrosome were frequently observed (Figure 3B). Weakly TBCD-expressing cells showed two individual green fluorescent protein (GFP) spots, which we localized to regions on the centrioles at which γ-tubulin was also more abundant, presumably the proximal region [26] (Figure 3C).

Interestingly, we also noted that both the GFP–TBCD_888–1200_ and the longer GFP–TBCD_324–1200_ segments decorated the microtubules 48 h after transfection (Figure 3D). This finding suggests that these TBCD regions contain both a centriolar-targeting sequence and a microtubule/tubulin-binding domain. TBCD binding to microtubules has also been reported for Alp1p, the S. pombe orthologue [17]. However, there is so far no biochemical evidence that mammalian TBCD also binds polymerized tubulin. To investigate this point, we performed classical biochemical microtubule polymerization binding assays with in-vitro-synthesized radioactive HsTBCD and BtTBCD, but none of these studies confirmed that TBCD binds to microtubules. We also investigated this subject in vivo, using the transitory transfection of TBCD-encoding constructs in taxol-treated HeLa cells. However, we again failed to confirm that TBCD binds to microtubules. In conclusion, these results suggest that although TBCD contains a microtubule/tubulin-binding domain, it does not bind microtubules. CPAP, a protein required for centriole duplication [30], has also been reported to contain a microtubule-binding domain, but as observed with TBCD, it does not bind microtubules [31]. Therefore, we hypothesize that this microtubule/tubulin-binding region is necessary to drive TBCD and tubulins to the procentriolar structures.

Finally, we also observed that the TBCD fragments discussed above localized at the Fleming bodies (Figure 3D) during cytokinesis, and aberrant midbodies with increased lengths and decreased thicknesses were often observed (Figure 3E, open arrow). Another frequent finding in weakly expressing cells was an increased distance between centrioles (>2 µm; Figure 3E).

### TBCD Is Required for Mitotic-Spindle Organization and Spindle-Pole Cohesion

**Figure 4:**
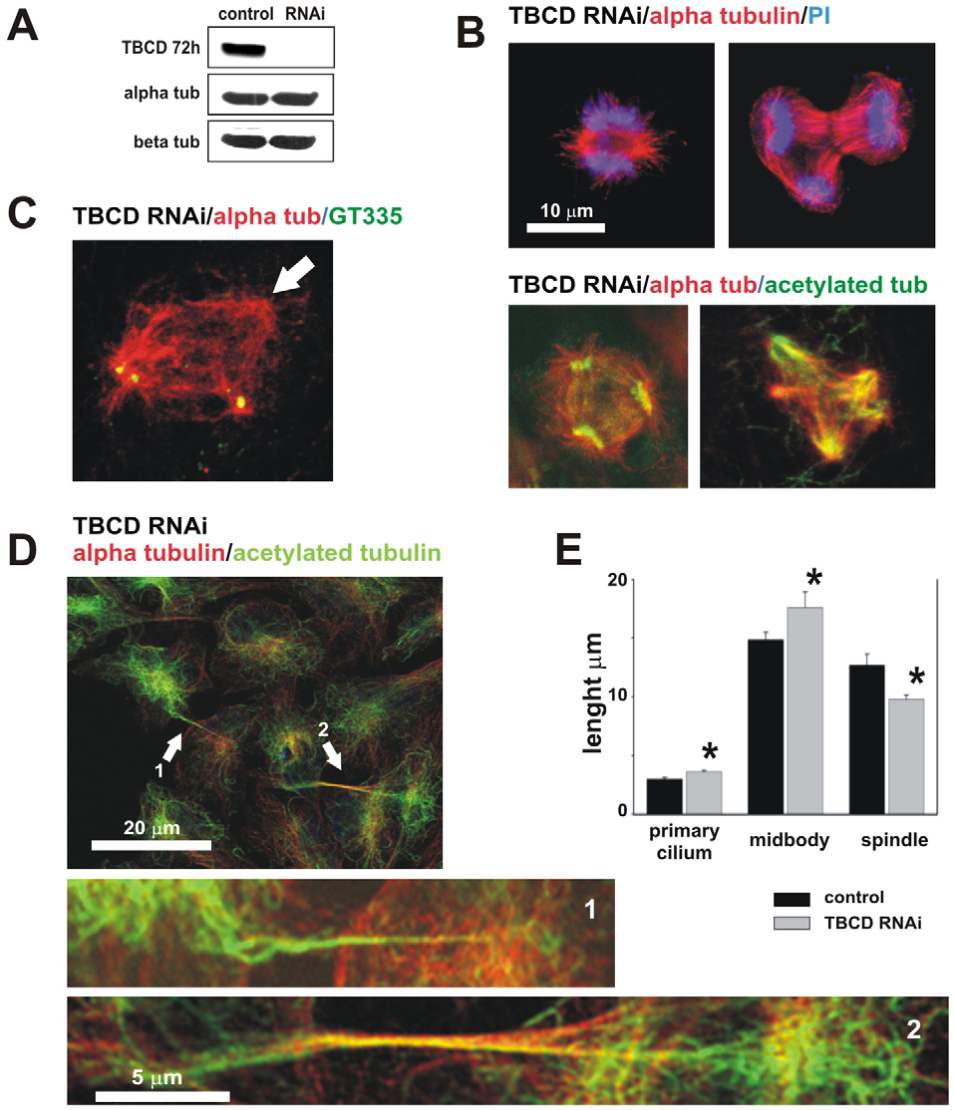
TBCD depletion resulted in microtubule spindle abnormalities and failure of cell abscission. (A) Western blot confirmation of TBCD silencing 72 h after siRNA treatment. Whole cell lysates (50 µg/lane) were loaded and analysed by immunoblotting with antibodies directed against CoD and the α- and β-tubulins. TBCD depletion did not affect α- or β-tubulin levels. (B) Confocal-microscopic projected images of different mitotic spindle defects observed after TBCD interference in HeLa cell cultures. Mitotic aberrations included abnormally short spindles, abnormal anaphase figures, and multipolar spindle defects, among others. (C) Acentriolar spindle poles were also observed (arrow). (D) TBCD silencing resulted in cell abscission failure. The maintenance of cytoplasmic bridges containing microtubules is shown (1, 2). These abnormally long midbodies are characterized by their content of acetylated microtubules (2). (E) Statistical analysis of the length of the primary cilia (DF = 147; P = 3×10−3) and midbodies (DF = 174; P = 0.1) confirmed significant increases in the lengths of these two structures after TBCD depletion. A highly significant reduction in the spindle pole-to-pole distance (DF = 63; P = 5×10−4) was also detected (*).

There is considerable evidence in the literature suggesting a role for TBCD in mitosis at metaphase, but no mechanism has yet been established. To understand the role of this cofactor in mitosis, we silenced TBCD gene expression in HeLa cells with interference RNA. We used a pool of four synthetic RNAs designed for the knockdown of the HsTBCD gene, with guaranteed high specificity and a reduced off-target effect. We investigated TBCD expression by western blotting at 24, 48, and 72 h after interference. We observed that by 72 h, there was almost complete depletion of this protein in HeLa cell extracts, whereas both α- and β-tubulin levels were apparently unaffected (Figure 4A).

We observed 25% of aberrant mitotic figures and several kinds of spindle defects were common, such as unipolar, multipolar, disorganized, and abnormally short spindles (Figure 4B). Acentriolar spindle poles and mislocalized centrioles were also often observed (Figure 4C). We also noted that existing primary cilia looked more developed in the TBCD-silenced cultures. We quantified the pole-to-pole lengths of the mitotic spindles and primary cilia on confocal-microscopic projected images. Statistical analysis of the data confirmed a highly significant reduction in the spindle size (degrees of freedom [DF] = 63; P = 5×10−4) and a significant increase in the lengths of cilia (DF = 147; P = 3×10−3; Figure 4E). Longer primary cilia might reflect a G1 mitotic block, reported when changes resulted in centrosomal damage [29]. However, this finding could also indicate that TBCD plays a role in cilia disassembly, for instance by microtubule depolymerization.

### TBCD Participates in Microtubule Retraction at the Midbody and Cell Abscission

TBCD silencing also resulted in anaphase defects and aberrant microtubule arrays at the midzone (Figure 4B). However, more importantly, we observed that at cytokinesis, the midbodies were more persistent in the TBCD-silenced cells than in the controls. We observed cells, apparently at interphase, that were still connected to sister cells by long, narrow, and continuous cytoplasmic bridges containing acetylated microtubules (Figure 4D). We measured the sizes of the midbodies on confocal-microscopic projected images and confirmed by statistical analysis that there was a significant difference in the average lengths of these structures in cultures where TBCD had been silenced (DF = 174; P = 0.1). This finding demonstrates that the localization of TBCD at the midbody is genuine. However, more interestingly, persistent microtubules at the midbody suggest that TBCD plays a role in microtubule retraction during cell abscission, probably by involvement in tubulin heterodimer dissociation.

### TBCD Is Recruited into “Centriolar Rosettes” during Ciliogenesis

The biogenesis of nascent centrioles is tightly coupled to the cell cycle and is precisely co-ordinated with DNA replication [32]. However, in differentiating ciliated cells, this finely tuned mechanism controlling the one-mother-one-daughter centriole ratio is overcome to allow the assembly of the masses of centrioles that become basal bodies. During early ciliated-cell differentiation, up to nine daughter centrioles assemble simultaneously, either around one maternal template or in association with a matrix of electron-dense fibrous granules, called a “deuterosome” [33], [34]. These pre-centriolar structures have been called “centriolar rosettes” because of their peculiar flower-petal configuration [32], [35], [36]. After assembly, the centrioles migrate towards the apical pole of the epithelial cell and become basal bodies. In mammals, ciliated epithelium is found in the airways, the oviduct, and in the ependymal layer of the brain. It is widely accepted that the centrioles formed in ciliated cells and the centrosomes in dividing cells have similar protein constituents [37]. Therefore, we investigated the function of TBCD during ciliogenesis, using the ependymal epithelium of the brain as a model system.

**Figure 5:**
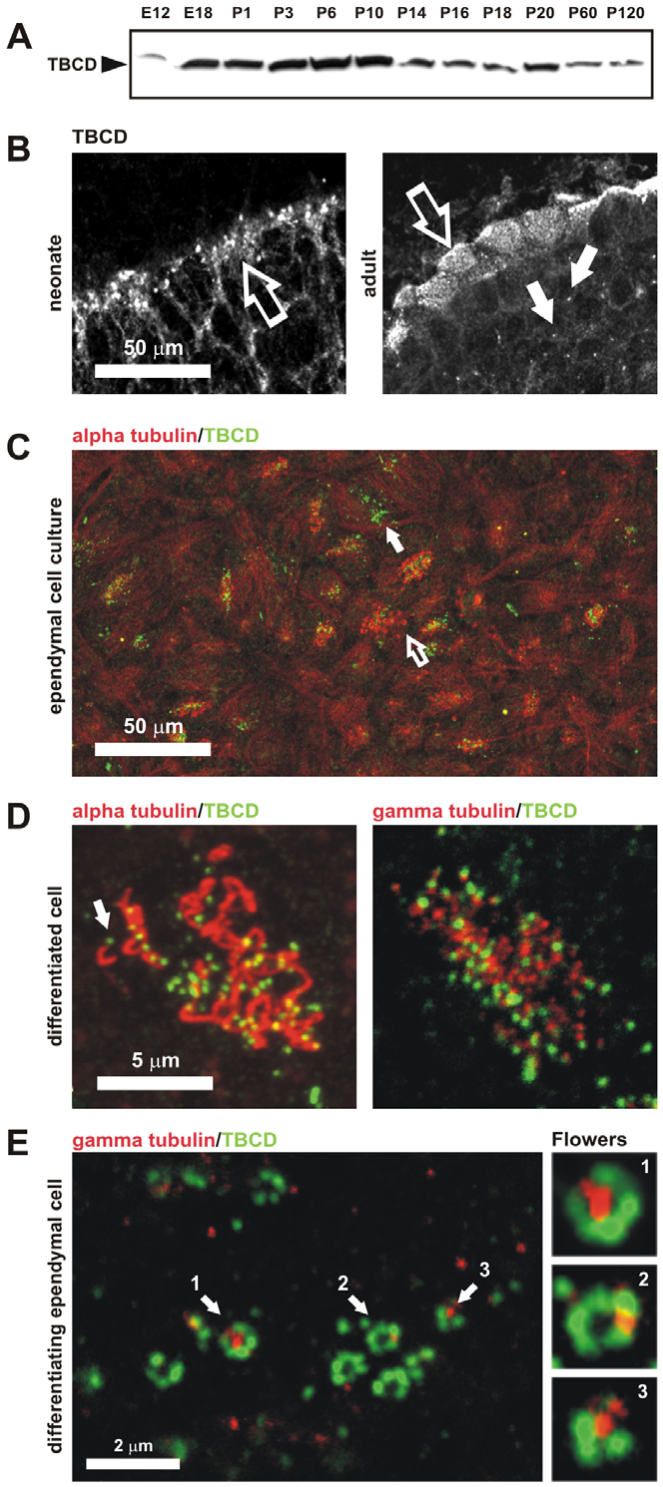
TBCD is required in ciliogenesis. (A) TBCD is abundantly expressed during neurogenesis. Western blot of total protein extracts (50 µg/lane) from developing and adult mouse brains obtained at the indicated embryonic and postnatal ages. (B) Confocal-microscopic images of cryo-sections of neonatal and adult murine cerebral ependymal epithelium immunostained for TBCD. This cofactor accumulated in clusters localized in the ependymal cell cytoplasm (left, open arrow). In the adult, TBCD localized at the apical borders of the ependymal cells, where the basal bodies are located (right, open arrow). TBCD was also detectable on parenchymal cell centrosomes (solid arrow). (C) A general view of an ependymal cell primary culture in which cilia, immunostained with anti-tubulin antibody, and TBCD are labelled. These culture conditions allow the observation of epithelial cells at various differentiation stages (I–IV, see the text). (Solid arrow) TBCD accumulated in differentiating cells with no cilia, whereas ciliated cells (open arrow) contained fewer TBCD spots. (D) Detail of the apical borders of differentiated ependymal cells where a single TBCD spot was observed at the base of each cilium (arrow). These TBCD spots partially co-localized with the γ-tubulin-labelled basal body. (E) Detail of the cytoplasm of a differentiating ependymal cell (stages I–II), where the assembly of several centriolar rosettes, labelled for TBCD and γ-tubulin, are shown. TBCD accumulated into structures of approximately 0.3 µm in diameter, which created ring-like structures that were often associated with a single γ-tubulin spot of a similar diameter (1, 2, and 3).

To confirm the presence of detectable amounts of TBCD and to study the pattern of protein expression during neurogenesis, we first investigated TBCD expression by western blotting in total murine brain extracts obtained at different ages (Figure 5A). The region of the brain in which TBCD was most expressed was localized by immunohistochemistry. As shown in Figure 5B, TBCD immunostaining was identified at the ependymal epithelia. In neonates, the signal was localized in large aggregates within the cytoplasm of differentiating ependymal cells, whereas in adults, small TBCD spots were concentrated at the apical borders of these cells, apparently co-localizing with the basal bodies. TBCD immunostaining was also observed at the centrosomes of surrounding parenchymal cells (Figure 5B, solid arrows).

**Figure 6:**
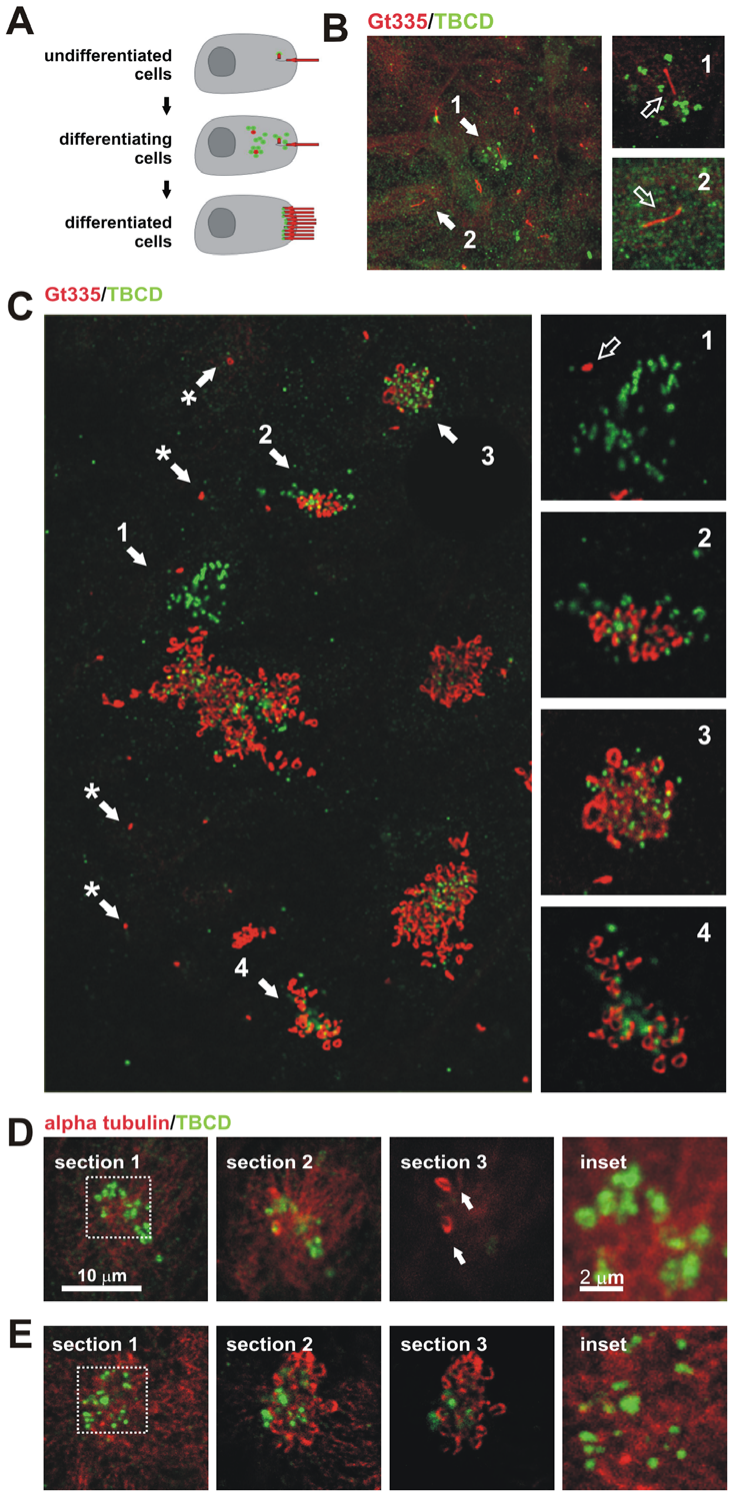
TBCD changes during ciliogenesis in ependymal cells. (A) Diagram of the location of TBCD accumulation (green) with respect to the centrioles and cilia (red) during ependymal cell differentiation. (B) Short-term cultures display cells at stages I and II, where only a single cilium per cell is observed, presumably the primary cilium. (1) The presence of the primary cilium is often accompanied by the development of small cytoplasmic TBCD clusters containing 1–3 TBCD spots of approximately 0.3 µm. (2) Uncommitted epithelial cells in the culture displayed diffuse TBCD immunostaining. This confocal plane did not coincide with the daughter centriole. (C) General view of a culture immunostained with GT335 and for TBCD. Here, the relationship between TBCD and the assembly of ependymal cilia can be seen. (*) Primary cilia with their corresponding TBCD-labelled centrioles are observed. (1) Differentiating cells (stages I–II) contain TBCD aggregates in their cytoplasm (Figure 5E). (2–4) Cilia assembly occurs after TBCD spots migrate to the apical border of the cells as single spots, presumably accompanying newly developed centrioles. (D-E) A series of confocal sections obtained from the apical borders of the epithelial cells towards the cilia. Cells were immunostained with anti-α-tubulin and anti-TBCD antibodies to show the relationships between this cofactor, the microtubule cytoskeleton, and the cilia. (D) Differentiating cells displaying a few cilia (arrows) show TBCD clusters visible in the cytoplasmic confocal planes (inset). (E) Differentiated cells containing several cilia show smaller TBCD aggregates in the confocal planes, which coincide with the basal bodies of existing cilia (inset).

These results led us to develop a primary cell culture method that would allow the detailed study of the full differentiation process of ependymal cells in vitro (Materials and Methods; Figure 5C, Figure 6). A similar culture model developed by Vladar and Stearns [37] describes four stages (I–IV) of tracheal epithelial cell differentiation. In stage I, centrosomal proteins accumulate at the centrosome; in stage II, primary cilia are removed and centrosomal proteins accumulate in clusters; finally, in stages III and IV, the centrioles move towards the plasma membrane for the assembly of ciliary axonemes. Although the cells in our culture system were not synchronized, we observed a perfect correlation between these stages and the differentiation processes of individual ependymal cells.

The examination of immunostained ependymal cell cultures (stages III–IV) revealed that TBCD expression was more abundant in undifferentiated and differentiating ependymal cells (Figure 5C, solid arrow). In differentiated multiciliated cells, TBCD was restricted to small dots of approximately 0.3 µm in diameter that were localized at the base of each cilium (Figure 5D, arrow). Co-localization with γ-tubulin was only partial in the basal bodies, thus confirming previous electron-microscopic observations (Figure 1D). Interestingly, we found that differentiating cells in these cultures displayed cytoplasmic TBCD clusters of approximately 0.3 µm in diameter (stage II), consistent with the size of centrioles, which were frequently organized into C-like or ring-like structures of approximately 1 µm in diameter (Figure 5E). Double immunostaining of these cultures with anti-γ-tubulin antibody revealed that some of these conformations contained a single central spot of γ-tubulin of approximately 0.3 µm in diameter, surrounded by several (4–6) TBCD aggregates. This structure, with a flower-petal configuration, is identical to the so-called procentriolar “rosettes” described in other systems, in which massive centriologenesis is triggered by different means. Therefore, we believe that these results constitute a solid argument supporting a role for TBCD in centriologenesis under physiological conditions.

This culture system served to further support findings on HeLa cells confirming TBCD localization at the base of the primary cilium in undifferentiated neuroepithelial cells (stages I–II; Figure 6).

## Discussion

Since the discovery of TBCs, research into them has largely focused on tubulin biogenesis. This study implicates TBCD in several crucial cell processes. We have shown that TBCD is concentrated at the centrioles and basal bodies, where it plays roles in centriologenesis, ciliogenesis, and spindle organization. TBCD is also found at the Fleming bodies, ring-like structures localized at the midbody during cytokinesis, where it is implicated in cell abscission. In view of all the existing biochemical data, these findings must be interpreted in the context of tubulin-related processes, such as tubulin supply or dissociation.

This study demonstrates for the first time TBCD accumulation in developing centrioles during physiological or induced centriologenesis in different systems. TBCD accumulates in procentrioles and is gradually lost during the maturation process, coinciding with microtubule glutamylation, a post-translational modification of tubulin leading to greater microtubule stability. Therefore, we hypothesize that TBCD participates in the supply of tubulin to procentrioles, while binding and protecting the developing centriolar blades until microtubule assembly is complete.

We have also documented the recruitment of this cofactor to the Fleming body during cytokinesis, where TBCD plays a role in cell abscission. However, this is not a novel phenomenon, but has already been observed in plants [19]. Cell abscission requires the co-ordination of two key processes, membrane supply and microtubule retraction. We believe that because this cofactor is an efficient depolymerizing protein [6], [8], it must contribute to the disassembly of the microtubules at the midbody during cell separation. Remarkably, there are already established molecular links between the centrosome and midbody for other proteins, which are predominantly involved in the process of membrane supply [38]. Here, we have shown that not only TBCD, but also the γ- and ε-tubulins occur at the Fleming body. Although we cannot explain the role of ε-tubulin in this structure, we believe that TBCD and γ-tubulin play a shared role in this secondary MTOC during cytokinesis. Significantly, a failure of cell abscission has also been reported in cultures in which γ-tubulin had been silenced [39].

Our data also demonstrate a significant reduction in the pole-to-pole spindle length after TBCD depletion, suggesting that this cofactor participates in microtubule spindle dynamics. Indeed, F16D3.4, the C. elegans TBCD orthologue, has been shown to be a regulator of the microtubule growth rate [21]. Classical experiments performed by Mitchinson [40] and later reviewed by Rogers et al. [41] demonstrated that spindle microtubules are extremely dynamic, insofar as tubulin subunits are continuously incorporated into the microtubule plus ends while they are removed at their minus ends, embedded at the spindle poles. This model implies that there should be identical tubulin polymerizing–depolymerizing rates at both microtubule extremes to maintain the spindle symmetry and length. At the minus ends, proteins such as Kin 1 or katanin could be pulling and severing the microtubules, whereas at the plus ends, there must be a sufficient tubulin supply to maintain a constant microtubule length. Both mechanisms require an exquisite co-ordination of tubulin processing and trafficking events between microtubule extremes, where we hypothesize that TBCD is implicated. Our data show how excess TBCD releases microtubules from the centrosome and produces γ-tubulin-containing supernumerary MTOCs at metaphase, findings suggesting that TBCD contributes to microtubule release from the centrosome during mitosis, as well as in interphase. Although the molecular mechanism of microtubule release from the centrosome is still unknown, it is broadly accepted that the anchoring of the microtubule to this organelle depends on the γ-tubulin ring complex (γTuRC), and that any mechanism leading to γTuRC detachment releases microtubules into the cytosol. TBCD is one of several candidate proteins that may anchor the γTuRC to the centrosome [24]. The contribution of TBCD to this process would explain why in mammalian centrioles, microtubules are nucleated through their entire length (where there is γ-tubulin), whereas microtubules are only anchored in the distal part of the mother centriole [42], a region devoid of TBCD.

Centrioles are not just required at the centrosome but also for the assembly of the basal bodies of cilia. Unfortunately, the mechanisms involved in massive centriologenesis and centriolar differentiation into basal bodies are still unclear. Recent studies have revealed that more than 1,000 different proteins are implicated in ciliary function, but almost no information regarding their roles in centriolar differentiation or ciliary axoneme assembly is available. Here, we have shown that TBCD is also crucial in centriologenesis via the acentriolar pathway, where it is organized into “centriolar rosettes”. We propose a model in which TBCD molecules associate to form a scaffold for the assembly of other centriolar proteins, in both the canonical and the acentriolar assembly pathways. Further studies of TBCD at the biochemical level will shed light on the mechanisms underlying the way this tubulin cofactor is recruited into these procentriolar structures during the differentiation of multi-ciliated cell. An increased understanding of the process of ciliogenesis will assist the better diagnosis and treatment of cilia-associated human diseases, such as primary ciliary dyskinesia and Bardet–Biedl, Alstrom, and Meckel–Gruber syndromes.

## Materials and Methods

### Ethics Statement

25 Swiss-Webster mice were used in this study. They were killed according to PHS policy and the U.S. National Institutes of Health guidelines.

### Antisera, Immunocytochemistry, Flow Cytometry and Cultures

Affinity purified primary antibodies were produced against TBCD recombinant protein fragments of BtTBCD (obtained from Dr. Cowan, New York University, NY, USA), HsTBCD isoform 1 and M. musculus TBCD (RPZD, Berlin, Germany). Rabbit sera were affinity purified against HsTBCD baculovirus-infected Sf9 cell extracts, as described previously [43]. CHO cell (ATCC^R^ number: CCL-61™) S-phase arrest was induced by treatment with 2 mM thymidine (Sigma-Aldrich, MO, USA) for 16 h. Immuno-electron-microscopic analysis was performed on 80 nm sections of 4% paraformaldehyde/0.1% glutaraldehyde-fixed tissue embedded in Unicryl (BBInternational, Cardiff, UK). Antibodies used were anti-α- and anti-β-tubulin (B512 and Tub2.1, respectively), anti-acetylated tubulin, anti-NEDD1/GCP-WD, and anti-ε- and anti-δ-tubulin antibodies (all from Sigma Aldrich). The anti-glutamylated tubulin antibody (GT335) was a gift from Dr Janke (CNRS, Montpellier, France) and the anti-γ-tubulin antibody (TU30) from Dr Draber (Institute of Molecular Genetics, Prague, Czech Republic). The GFP-centrin1 construct was kindly provided by Dr M. Bornens (Institut Curie, Paris). Secondary antibodies were Alexa-Fluor-488-conjugated goat anti-rabbit IgG and goat-anti-mouse IgG, Alexa-Fluor-647-conjugated goat anti-mouse IgG (Molecular Probes, Invitrogen), Cy3-conjugated goat anti-mouse IgG and goat anti-mouse IgG1, and Cy5-conjugated goat anti-rabbit IgG (Jackson ImmunoResearch Laboratories, Inc.) and a 10 nm anti-rabbit IgG gold conjugate (Sigma, St. Louis, MO). RNA interference was performed with a “Smart Pool” mixture targeting human TBCD (Dharmacon, CO, USA). TBCD silencing was confirmed 24, 48, and 72 h after RNAi treatment by western blotting. TBCD cDNA fragments were produced by PCR. The TBCD mutants were sequenced and cloned into the pEGFP-C2 and/or pEGFP-N3 vectors from Invitrogen (Life technologies, California USA). P2 cerebellar mixed primary cell cultures were grown on poly-L-lysine and laminin-coated (Sigma-Aldrich, MO, USA) coverslips and were grown in 10% foetal bovine serum in IMDM medium (all from Gibco, Invitrogen). The cultures were fixed at different time points after plating (after 5–20 days in culture). Beating cilia were observed approximately 10 days after dissociation. Cell lines and cultures used for immunocytochemistry were fixed in ice-cold (−20°C) methanol or 4% paraformaldehyde, and further permeabilized in phosphate-buffered saline (PBS)–0.1% Triton X-100 in PBS (PBS-T). For some experiments microtubules were depolymerized with 2 µM nocodazole and cold (4°C) treatments for 30 min. CHO cell centrosome overduplication was induced by treatment with 2 mM thymidine (Sigma-Aldrich) for 16 h. Flow cytometry was performed on cells with Hoechst-stained DNA 15 and 30 h after transfection with HsTBCD–YFP or H2B–YFP kindly supplied by Dr DiCroce (ICREA, CRG, Barcelona, Spain) as the control. A Becton Dickinson FACS CantoII equipment equipped with a 405 nm and 488 nm laser diodes was used. Ten thousand cells were collected for each sample. The data were analysed using the FACS Diva software (Becton Dickinson, NJ, USA).

### Confocal Microscopy, Measurements and Statistical Analysis

Cell measurements were made on confocal-microscopic projected images obtained with a Zeiss 63× lens. Microscopy was performed on a Zeiss LSM-510 confocal microscope equipped with an argon (488 nm) laser and two HeNe (543 and 633 nm) lasers. Images in the same confocal planes were scanned sequentially to avoid fluorescent channel emission cross-talk. The maximum confocality was according to the manufacturer's instructions. A t test was performed on the data obtained for confocal microscopy measurements on two different coverslips, resulting in three different experiments. Statistical analysis of data and graphing were performed using the SigmaPlot 8.0 software (Systat Software, Richmond, CA). The data in Figure 4E represent mean values and standard error bars.

### Microtubule Binding Tests

Bovine brain tubulin was purified as described by [44]. Full-length cDNAs encoding HsTBCD and BtTBCD were used as the templates for coupled transcription and translation in rabbit reticulocyte cell-free lysates (Promega Corporation) in the presence of ^35^S-methionine (>1000 Ci/mmol; Amersham Pharmacia Biotech), as described elsewhere [45]. The products of in vitro TBCD synthesis were incubated with taxol-stabilized microtubules, as previously described [8].

## Supporting Information

Figure S1 Specificity of TBCD antibodies and confocal images at metaphase. (A) Specificity of the antibodies directed against TBCD produced for the study. Western blot of total HeLa cell extracts (50 µg/lane) immunostained with an antibody recognizing full-length HsTBCD (antibody 1) and a second antiserum recognizing a fragment of mouse TBCD (MmTBCD; antibody 2). Both antibodies demonstrated great specificity after affinity purification. (B) Confocal-microscopic images of triply/doubly labelled HeLa cells at metaphase. TBCD partially co-localized with NEDD1/GCP-WD but did not co-localize with ε-tubulin.
(0.88 MB TIF)

Figure S2 TBCD is associated with centriologenesis in different systems. (A) TBCD immunostaining in thymidine-treated S-arrested CHO cells. (top) CHO cells generally contained aberrant centriolar numbers and when blocked at S-phase by thymidine treatment, underwent repeated cycles of centriole synthesis. After 16 h blockage, TBCD accumulation at the developing centrioles was specifically observed. (B) TBCD immunostaining of a HeLa cell transfected with a construct encoding PLK4. This protein is known to trigger centriologenesis when overexpressed. TBCD labelled the developing centrioles 24 h after transfection.
(1.31 MB TIF)

## References

[1] Andersen JS, Wilkinson CJ, Mayor T, Mortensen P, Nigg EA (2003). Proteomic characterization of the human centrosome by protein correlation profiling.. Nature.

[2] Lewis SA, Tian G, Cowan NJ (1997). The alpha- and beta-tubulin folding pathways.. Trends Cell Biol.

[3] López-Fanarraga M, Avila J, Guasch A, Coll M, Zabala JC (2001). Review: postchaperonin tubulin folding cofactors and their role in microtubule dynamics.. J Struct Biol.

[4] Szymanski D (2002). Tubulin folding cofactors: half a dozen for a dimer.. Curr Biol.

[5] Tian G, Lewis SA, Feierbach B, Stearns T, Rommelaere H (1997). Tubulin subunits exist in an activated conformational state generated and maintained by protein cofactors.. J Cell Biol.

[6] Bhamidipati A, Lewis SA, Cowan NJ (2000). ADP ribosylation factor-like protein 2 (Arl2) regulates the interaction of tubulin-folding cofactor D with native tubulin.. J Cell Biol.

[7] Kortazar D, Carranza G, Bellido J, Villegas JC, Fanarraga ML (2006). Native tubulin-folding cofactor E purified from baculovirus-infected Sf9 cells dissociates tubulin dimers.. Protein Expr Purif.

[8] Martín L, Fanarraga ML, Aloria K, Zabala JC (2000). Tubulin folding cofactor D is a microtubule destabilizing protein.. FEBS Lett.

[9] Abruzzi KC, Smith A, Chen W, Solomon F (2002). Protection from free beta-tubulin by the beta-tubulin binding protein Rbl2p.. Mol Cell Biol.

[10] Archer JE, Vega LR, Solomon F (1995). Rbl2p, a yeast protein that binds to beta-tubulin and participates in microtubule function in vivo.. Cell.

[11] Archer JE, Magendantz M, Vega LR, Solomon F (1998). Formation and function of the Rbl2p-β-tubulin complex.. Mol Cell Biol.

[12] Fanarraga ML, Párraga M, Aloria K, del Mazo J, Avila J (1999). Regulated expression of p14 cofactor A during spermatogenesis.. Cell Motil Cytoskel.

[13] Bartolini F, Tian G, Piehl M, Cassimeris L, Lewis SA (2005). Identification of a novel tubulin-destabilizing protein related to the chaperone cofactor E.. J Cell Sci.

[14] Keller CE, Lauring BP (2005). Possible regulation of microtubules through destabilization of tubulin.. Trends Cell Biol.

[15] Kortazar D, Fanarraga ML, Carranza G, Bellido J, Villegas JC (2007). Role of cofactors B (TBCB) and E (TBCE) in tubulin heterodimer dissociation.. Exp Cell Res.

[16] Hoyt MA, Stearns T, Botstein D (1990). Chromosome instability mutants of Saccharomyces cerevisiae that are defective in microtubule-mediated processes.. Mol Cell Biol.

[17] Hirata D, Masuda H, Eddison M, Toda T (1998). Essential role of tubulin-folding cofactor D in microtubule assembly and its association with microtubules in fission yeast.. EMBO J.

[18] Liu CM, Meinke DW (1998). The titan mutants of Arabidopsis are disrupted in mitosis and cell cycle control during seed development.. Plant J.

[19] Steinborn K, Maulbetsch C, Priester B, Trautmann S, Pacher T (2002). The Arabidopsis PILZ group genes encode tubulin-folding cofactor orthologs required for cell division but not cell growth.. Genes Dev.

[20] Sönnichsen B, Koski LB, Walsh A, Marschall P, Neumann B (2005). Full-genome RNAi profiling of early embryogenesis in Caenorhabditis elegans.. Nature.

[21] Srayko M, Kaya A, Stamford J, Hyman A (2005). A. Identification and characterization of factors required for microtubule growth and nucleation in the early C. elegans embryo.. Dev Cell.

[22] Fedyanina OS, Mardanov PV, Tokareva EM, McIntosh JR, Grishchuk EL (2006). Chromosome segregation in fission yeast with mutations in the tubulin folding cofactor D.. Curr Genet.

[23] Fedyanina OS, Book AJ, Grishchuk EL (2009). Tubulin heterodimers remain functional for one cell cycle after the inactivation of tubulin-folding cofactor D in fission yeast cells.. Yeast.

[24] Cunningham LA, Kahn RA (2008). Cofactor D functions as a centrosomal protein and is required for the recruitment of the gamma-tubulin ring complex at centrosomes and organization of the mitotic spindle.. J Biol Chem.

[25] Chang P, Stearns T (2000). Delta-tubulin and epsilon-tubulin: two new human centrosomal tubulins reveal new aspects of centrosome structure and function.. Nat Cell Biol.

[26] Bornens M (2002). Centrosome composition and microtubule anchoring mechanisms.. Curr Opin Cell Biol.

[27] Kleylein-Sohn J, Westendorf J, Le Clech M, Habedanck R, Stierhof YD (2007). Plk4-induced centriole biogenesis in human cells.. Dev Cell.

[28] Rodrigues-Martins A, Bettencourt-Dias M, Riparbelli M, Ferreira C, Ferreira I (2007). DSAS-6 organizes a tube-like centriole precursor, and its absence suggests modularity in centriole assembly.. Curr Biol.

[29] Mikule K, Delaval B, Kaldis P, Jurcyzk A, Hergert P (2007). Loss of centrosome integrity induces p38-p53-p21-dependent G1-S arrest.. Nat Cell Biol.

[30] Tang CJ, Fu RH, Wu KS, Hsu WB, Tang TK (2009). CPAP is a cell-cycle regulated protein that controls centriole length.. Nat Cell Biol.

[31] Hsu WB, Hung LY, Tang CJ, Su CL, Chang Y (2008). Functional characterization of the microtubule-binding and -destabilizing domains of CPAP & d-SAS-4.. Exp Cell Res.

[32] Tsou MF, Stearns T (2006). Mechanism limiting centrosome duplication to once per cell cycle.. Nature.

[33] Dirksen ER (1991). Centriole and basal body formation during ciliogenesis revisited.. Biol Cell.

[34] Hagiwara H, Ohwada N, Takata K (2004). Cell biology of normal & abnormal ciliogenesis in the ciliated epithelium.. Int Rev Cytol.

[35] Dawe HR, Farr H, Gull K (2007). Centriole/basal body morphogenesis and migration during ciliogenesis in animal cells.. J Cell Sci.

[36] Strnad P, Gönczy P (2008). Mechanisms of procentriole formation.. Trends Cell Biol.

[37] Vladar EK, Stearns T (2007). Molecular characterization of centriole assembly in ciliated epithelial cells.. J Cell Biol.

[38] Doxsey SJ (2005). Molecular links between centrosome & midbody.. Mol Cell.

[39] Shu HB, Li Z, Palacios MJ, Li Q, Joshi HC (1995). A transient association of gamma-tubulin at the midbody is required for the completion of cytokinesis during the mammalian cell division.. J Cell Sci.

[40] Mitchison TJ (1989). Polewards microtubule flux in the mitotic spindle: evidence from photoactivation of fluorescence.. J Cell Biol.

[41] Rogers GC, Rogers SL, Sharp DJ (2005). Spindle microtubules in flux.. J Cell Sci.

[42] Piel M, Meyer P, Khodjakov A, Rieder CL, Bornens M (2000). The respective contributions of the mother and daughter centrioles to centrosome activity and behavior in vertebrate cells.. J Cell Biol.

[43] Lajoie-Mazenc I, Tollon Y, Detraves C, Julian M, Moisand A (1994). Recruitment of antigenic gamma-tubulin during mitosis in animal cells: presence of gamma-tubulin in the mitotic spindle.. J Cell Sci.

[44] Ávila J, Soares H, Fanarraga ML, Zabala JC (2008). Isolation of microtubules and microtubule proteins..

[45] Zabala JC, Cowan NJ (1992). Tubulin dimer formation via the release of alpha- and beta-tubulin monomers from multimolecular complexes. Cell Motil.. Cytoskel.

