# Supplementary figures and images for: TBCD Links Centriologenesis, Spindle Microtubule Dynamics, and Midbody Abscission in Human Cells
Source: PLoS One. 2010 Jan 22;5(1):e8846. doi: 10.1371/journal.pone.0008846 (PMC2809749; doi:10.1371/journal.pone.0008846)

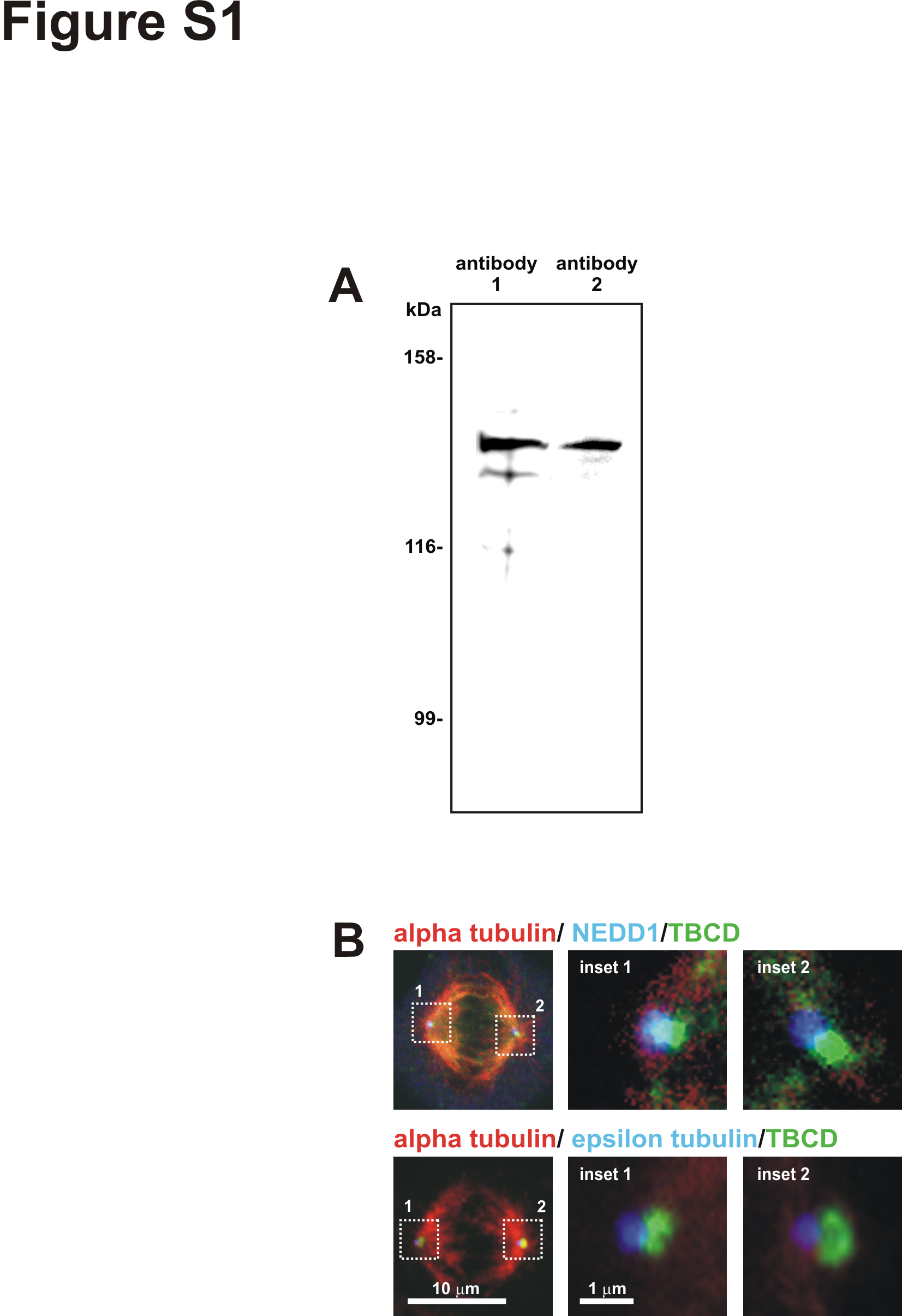

Supplement: Figure S1 — Specificity of TBCD antibodies and confocal images at metaphase. (A) Specificity of the antibodies directed against TBCD produced for the study. Western blot of total HeLa cell extracts (50 µg/lane) immunostained with an antibody recognizing full-length HsTBCD (antibody 1) and a second antiserum recognizing a fragment of mouse TBCD (MmTBCD; antibody 2). Both antibodies demonstrated great specificity after affinity purification. (B) Confocal-microscopic images of triply/doubly labelled HeLa cells at metaphase. TBCD partially co-localized with NEDD1/GCP-WD but did not co-localize with ε-tubulin. (0.88 MB TIF) [file pone.0008846.s001.tif]

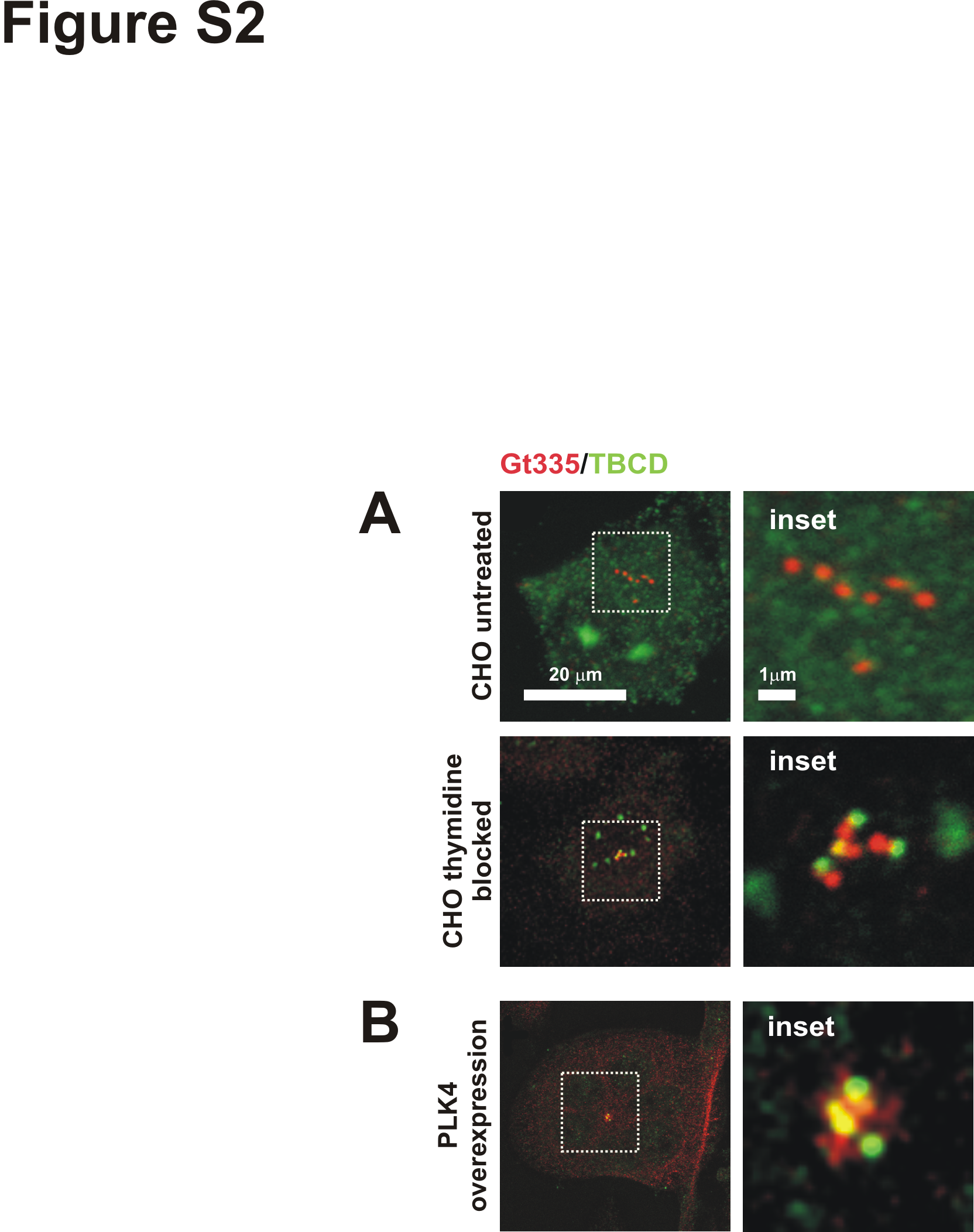

Supplement: Figure S2 — TBCD is associated with centriologenesis in different systems. (A) TBCD immunostaining in thymidine-treated S-arrested CHO cells. (top) CHO cells generally contained aberrant centriolar numbers and when blocked at S-phase by thymidine treatment, underwent repeated cycles of centriole synthesis. After 16 h blockage, TBCD accumulation at the developing centrioles was specifically observed. (B) TBCD immunostaining of a HeLa cell transfected with a construct encoding PLK4. This protein is known to trigger centriologenesis when overexpressed. TBCD labelled the developing centrioles 24 h after transfection. (1.31 MB TIF) [file pone.0008846.s002.tif]
